# Evaluating farmers’ perception toward the effectiveness of agricultural extension services in Ghana and Zambia

**DOI:** 10.1186/s40066-021-00325-6

**Published:** 2021-11-24

**Authors:** Albert Novas Somanje, Geetha Mohan, Osamu Saito

**Affiliations:** 1 United Nations University Institute for the Advanced Study of Sustainability, 5-53-70 Jingumae, Shibuya-ku, Tokyo, Japan; 2 Ministry of Agriculture, Lusumpuko House, Choma, Zambia; 3 Institute for Future Initiatives (IFI), The University of Tokyo, Tokyo, 133-8654 Japan; 4 Institute for Global Environmental Strategies (IGES), Kanagawa, 240-0115 Japan

**Keywords:** Pluralistic agricultural extension, Participatory approach, Performance indicators, Ghana, Zambia

## Abstract

**Background:**

In this study, we present the current situation and the role of agricultural extension services for farmers and indicates the potential solutions for the optimum effectiveness of these services. Thus, we investigate the vital determinants influencing the farmers’ attitudes toward using agricultural extension services in Ghana and Zambia.

**Methods:**

In this study, we used a mixed-method research analysis of data from a household survey of 240 farmers and 8 key informant interviews in the Upper West Region of Ghana and the Southern Province of Zambia.

**Results:**

The significant factors affecting the association of agricultural extension officers with farmers are regular meetings, demand for services and productivity, and the adoption rate of technology. Notably, approaches based on information communication technology indicators include owning cell phones; further, having radio access significantly affects agricultural practices. However, the role of gender, access to credit, and owning a television would influence food safety and nutrition.

**Conclusions:**

Understanding the critical determinants will provide potential solutions to national agricultural research institutes, private research entities, and policymakers to scale-up the effectiveness of agricultural extension services, particularly in Ghana and Zambia.

## Background

The Food and Agricultural Organization [1] projected the world population to reach 9 billion by 2050. Sub-Saharan Africa (SSA) has the fastest growing population and rate of urbanization globally, according to the United Nations Department of Economic and Social Affairs (UNDESA) [2]. The increasing population will also require more food production [1]. However, the increasing impact of urbanization, rural–urban migration, climate change, and poverty poses a challenge to food production [3–5]. Agricultural extension is critical to improving agricultural production, adaptation to climate change, poverty reduction, and food security [6–8].

Agricultural extension is a significant determinant that helps agricultural value chain actors such as farmers increase agricultural productivity, thereby improving income, alleviating poverty and enhancing food security. Empirical studies have shown that agricultural extension training is significant in agronomic practices, diet diversification and nutrition [9], sustainable agriculture [10], achieving sustainable development goals 1 (no poverty) and 2 (zero hunger) [11, 12], improving food security [12], and adopting advanced technologies [13]. The provision of agricultural services must adapt to the changing social, economic, and environmental indicators affecting the entire food system from production to consumption [6–8].

This study defines agricultural extension services (also referred to as agricultural advisory services) as the complete composition of organizational structures that facilitates and engages people in agricultural production, marketing, processing, and consumption and builds the capacity to improve their livelihoods [7, 14, 15]. Agricultural extension is essential in both the urban and rural agricultural value chain as a critical link for capacity building [7]. The entire agricultural value chain requires information and technology flow, and agricultural extension could contribute to the sustainability of urban–rural linkages [16]. Additionally, according to International Food Policy Research Institute (IFPRI) [5], it contributes to the attainment of sustainable development goals (SDGs) [5], specifically, SDGs 1 (no poverty), 2 (end hunger), 12 (sustainable production and consumption), 13 (climate change), 14 (life below water), and 15 (life on land) [15]. Both rural and urban agriculture are essential contributors to the food system [17]. Although urban agriculture is not a substitute for rural agriculture, it contributes to and supplements the environmental and human well-being in urban centers [17].

Delivering an excellent performance in agricultural extension services has been problematic due to rapid farming challenges and the historical setup of public agricultural extension services [18,19]. Public extension services have resulted in poor political and financial resources support [20]. Consequently, this has affected the sustainability of extension services in most developing countries, especially where donor support is limited [19]. It has been challenging to show the impact of extension in SSA because of the different models of extension and mixed evidence [21]. The extension system in developing countries functioned poorly in achieving its purpose and requires attention [6, 8]. Other scholars have argued that weak performance also results from the top-down approach that might not work accurately in areas that are distant to monitor, and accountability tends to be poor [22, 23].

Private agricultural extension services are an alternative to the public service system [24]. However, the private sector has used extension service delivery as a marketing tool, and they are more skewed to high-value agricultural services [25]. Notably, the public agricultural extension still has a significant role to play, e.g., where externalities for information and public goods are high [23, 26], such as environmental and conservation concerns [27], and the inclusion of disadvantaged groups, such as women who are significantly contributing to agricultural production [23, 26]. In assessing the gender gap in agricultural extension services in Ghana, Quaye et al. [26] indicate that specially targeted services are important for women in agriculture.

Several agricultural extension methods include the participatory approach, farmer field schools approach, training and visit approach, and farmer-to-farmer approach [18, 25, 27–32]. With the increasing use of and access to approaches based on information communication and technology (ICT), televisions, radios, and phones are used to complement the traditional extension methods [22, 31]. Pluralistic and participatory approaches focusing on facilitation (group formation) and technology (market access) use should perfectly complement SSA [21]. Kalusopa [22] finds that poor infrastructure and an unclear policy framework environment for using technology for agricultural extension service delivery to small-scale farmers in Zambia is challenging.

Generally, agriculture budgets significantly affect the effectiveness of agricultural extension, especially in building their capacity, but the funding has been significantly reduced [24, 25]. Literature has revealed that the capacity, such as resources, skills, efficiency, and extension staff, is lacking [22, 33, 34]. Moreover, the tertiary education system has not coped with the social, economic, and environmental needs of the required workforce [7], which is equally important. African countries agreed upon the improvement of the budget allocation to at least 10% of their national budget to agriculture for 5 years [35]. The progress on financial allocation to agriculture through the Comprehensive African Agricultural Development Programme framework has been unsatisfactory [36]. Africa and the sub-regions, on average, failed to meet the set target. Only a few countries managed to achieve the target in one or more fiscal years but not in every year [36] from 2003 to 2015 [37]. Of the 55 African Union countries, only 7 passed the 10% allocation for most years (Burkina Faso, Ethiopia, Guinea, Malawi, Mali, Niger, and Senegal). The most significant portion of the expenditure is on agricultural subsidies, whereas agricultural extension and research were lower in most countries [36].

In the literature, emphasis on farmers’ perceptions toward the effectiveness of agricultural extension service performance through extension approaches, such as participatory extension approach (PEA) training, is limited. In this study, PEA includes enhancing the productivity of agricultural livelihoods and well-being, motivating and mobilizing farmers’ participation in sustainable development, as well as empowerment through strengthening local organizations [25]. Sasidhar [38] identifies five reasons why agricultural PEA training is essential. First, training is an educational tool for modern challenges for people to help themselves. Second, training highlights the investment of human capital available for agricultural programs or project implementation. Third, training is a readily available tool for learning activities in agricultural extension. Fourth, training provides insights into the quality aspects in the development of training materials used by extension officers. Fifth, well-applied PEA training concepts and tools significantly affect the learners and their successful adoption of sustainable agricultural practices. Despite the highlighted vital point of training as an essential component in effective agricultural performance [8], effective practical implementation in most SSA countries remains challenging [24, 25].

Empirically, agricultural extension service performance is mostly assessed at an impact level, such as production, yield, and farmer’s profit margins, and not at a process level [39]. Birner et al. [8] contend that from an analytical perspective, measuring performance is more practical and less demanding than assessing impact. Furthermore, the study contributes to the comparative studies of the pluralistic agricultural extension service performance that is less explored in the literature. Specifically, we focus on pluralistic agricultural extension services using PEA in the Ministry of Food and Agriculture (MoFA), Ghana and the Ministry of Agriculture (MoA), Zambia [36, 40].

The pluralistic agricultural extension is the integrated approach to extension through different extension providers to farmers, such as the government, private sector, non-governmental organization and farmer-based community groups, such as cooperatives [8].

This study aims to assess (1) farmers’ perceptions toward the effectiveness of agricultural extension service performance and (2) the indicators influencing farmers’ perception, such as socioeconomic, multiple communication, and perceived return indicators. We discuss the policy implications of this study to enhance pluralistic agricultural extension performance through extension approaches that strengthen PEA training and the interaction between farmers and extension officers.

## Methods

### Area description

**Fig. 1:**
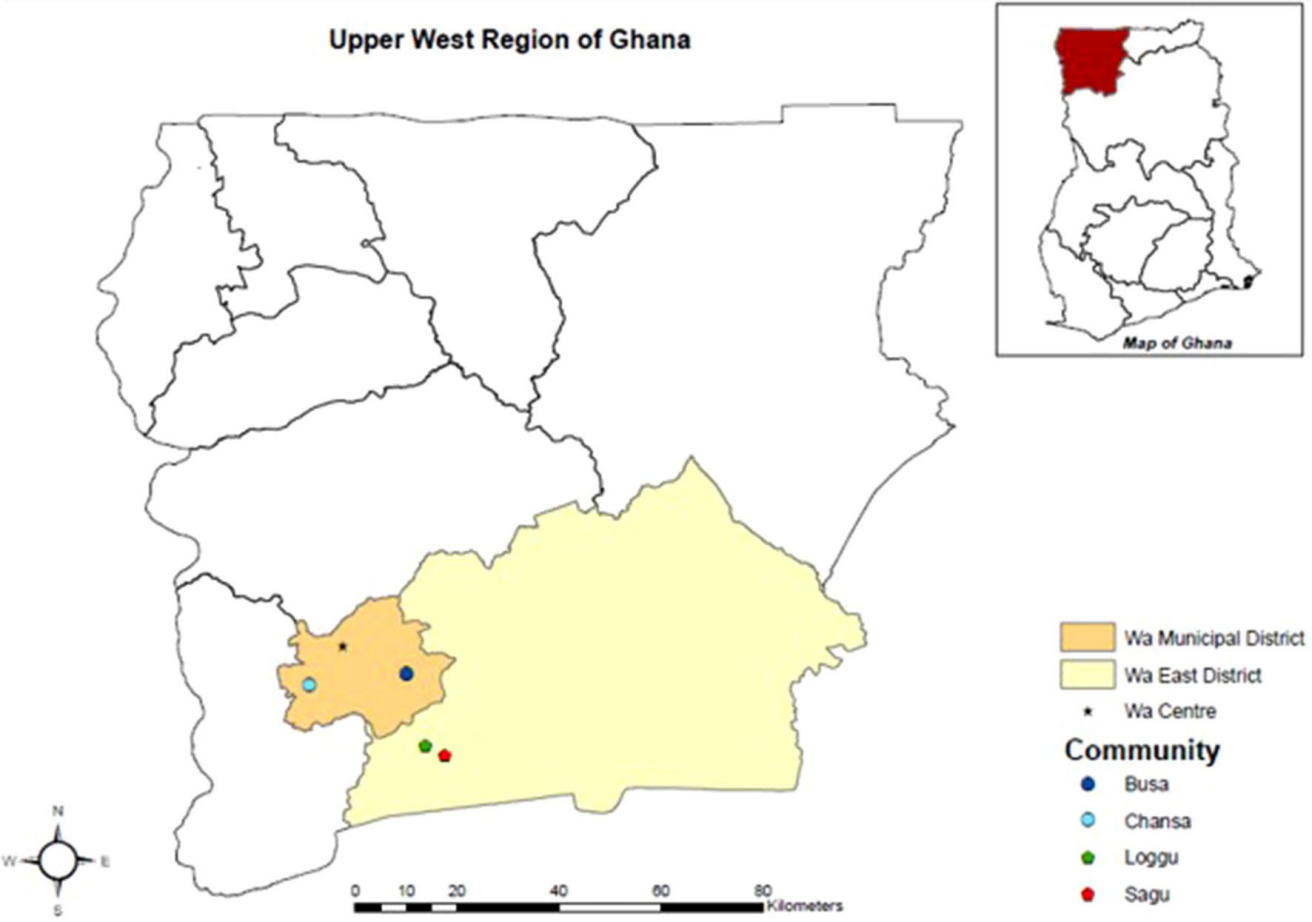
The study site in Wa Municipal and Wa East Districts of Ghana. Source: Field Survey (2019)

**Fig. 2:**
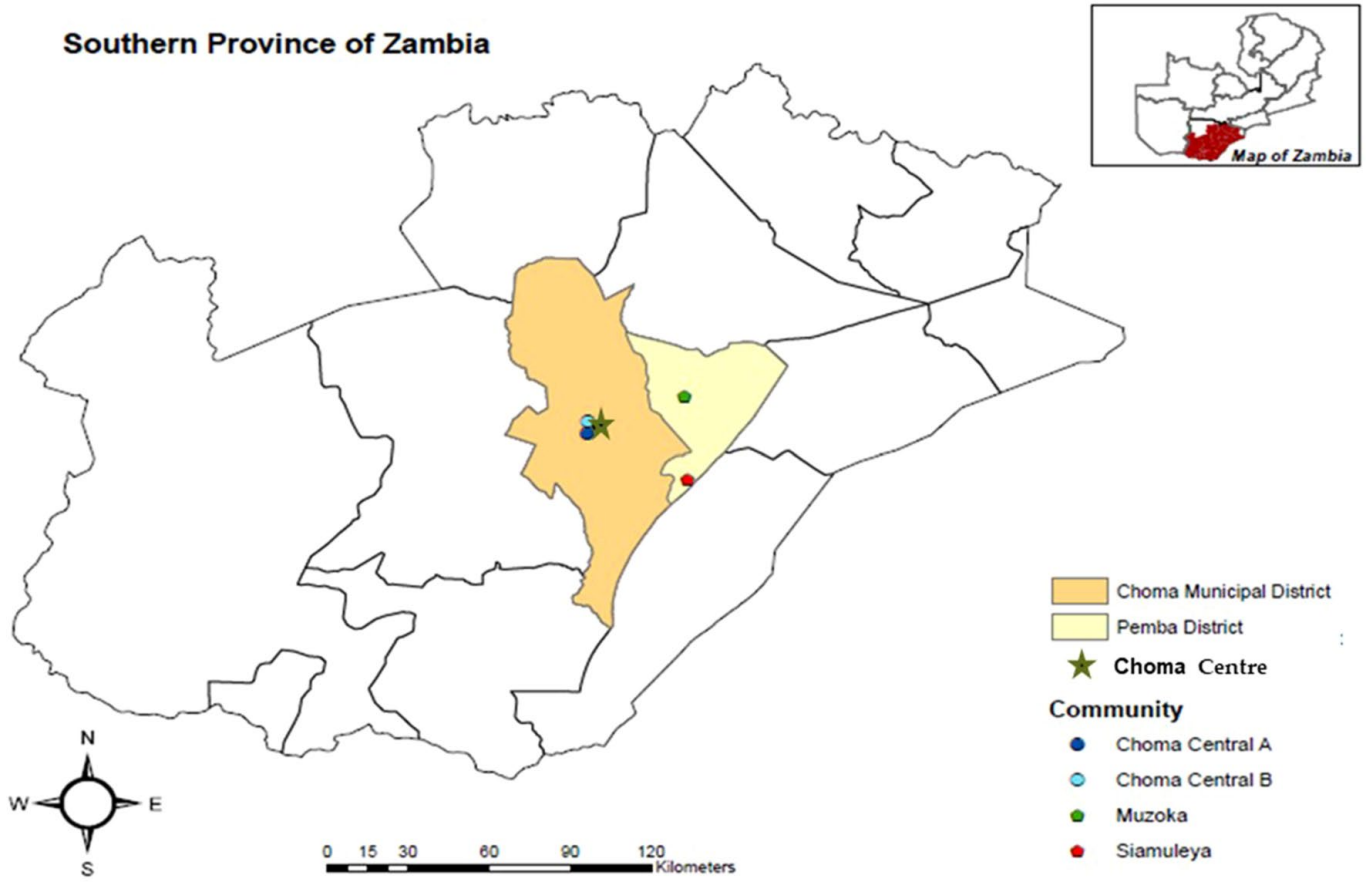
The study site in Choma Municipal and Pemba Districts in the Southern Province of Zambia. Source: Field Survey (2019)

Ghana and Zambia were purposively selected for this comparative study because they have a similar historical perspective [3] and a high rate of urbanization in SSA [2]. These two countries have a pluralistic agricultural extension system [40, 41]. Both the Upper West Region of Ghana (Fig. 1) and Southern Province of Zambia (Fig. 2) are faced by the challenge of negative net migration according to Central Statistical Office (CSO) [42] and Ghana Statistical Services (GSS) [43]. Furthermore, they are affected by climate impacts, especially in the agricultural sector [44–47].

This research was conducted in the Wa Municipal District (Bussa and Chansa communities), predominantly urban, and Wa East District (Loggu and Sagu communities), rural, in the Upper West Region of Ghana (Fig. 1) from January 22 to February 15, 2019. The Wa Municipal District is the regional capital of the Upper West Region. It has a population of 107,214 (49.4% male, 50.6% female), accounting for 66.3% urban, 33.7% rural, and 15.3% of the population of the Upper West Region [48]. The Wa Municipal District borders the Wa East District in the south, which is 100% rural with a population of 72,074 (50.5% male, 49.5% female), accounting for 10.3% of the population of the Upper West Region [48]. The two districts are predominantly Muslim [48], have one agricultural season, are the hottest and driest parts of the country, and have a predominantly coastal savanna agro-ecological zone with an annual rainfall of less than 1000 mm [47].

This research study was replicated in the Choma Municipal District (Choma Central A and B communities), predominantly urban, and Pemba District (Muzoka and Siamuleya communities), rural, in the Southern Province of Zambia (Fig. 2) from February 26 to March 23, 2019. According to the CSO [49], the Choma Municipal District has a population of 269,963, with 49% male and 51% female. The rural community accounts for 76% of the population, whereas the urban population is 24%. Choma is also the capital of the Southern Province, hosting all administrative offices for the region.

The Pemba District has a population of 71,802 (52.3% male, 48.7% female) and is predominantly rural [49]. In both districts, the predominant religion is Christianity. The Southern Province of Zambia has one agricultural season with extreme weather variability, is the hottest and driest part of the country, and falls in the agro-ecological region zone I that receives less than 800 mm of rain per year [44, 45].

### Research framework

**Fig. 3:**
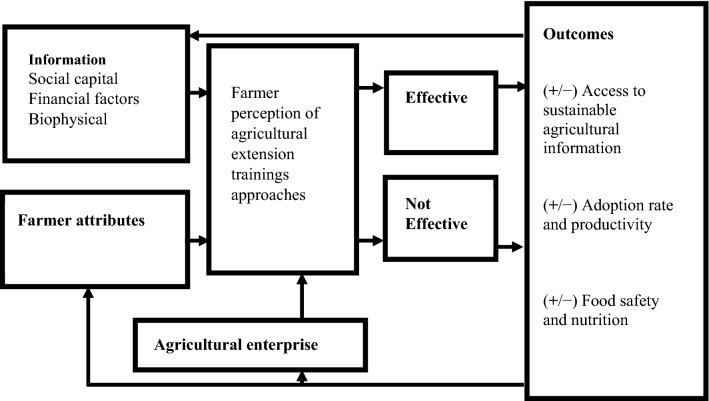
Conceptual framework. Source: Modified from Ntshangase et al. [52]

In this study, we focus on the descriptive assessment of the effectiveness of agricultural extension services that have been less emphasized in previous studies. To address the objective of this study, we integrated the framework for analyzing pluralistic agricultural extension performance through the effectiveness of information sources [8, 50] and perceptions [51, 52] based on the innovation diffusion theory by Rogers [53]. Information dissemination is the core mandate of agricultural extension services, innovation diffusion, and knowledge acquisition [52–54]. Birner et al. [8] and Swanson et al. [18] define effectiveness as meeting the objective or target set to deliver quality agricultural services through regular interaction with farmers, such as raising awareness and conducting meetings and visits. Farmers’ adoption and application of sustainable agricultural practices are also influenced by economic perspectives by having access to economic resources [52–54]. Adoption here is defined as farmers’ practical application of knowledge, information, or technology gained for potential productivity and agricultural sustainability [52]. Furthermore, the study indirectly measured food safety and nutrition knowledge as well as information impact using perception based on the need to integrate food safety and nutrition because they are closely related [55–57]. The socioeconomic indicators of the farmers also determine their perspectives on the interaction levels with agricultural extension personnel (Fig. 3). Some of the indicators identified in the literature include age, gender, education, household size, access to credit, land size, and farmers enterprising multiple communication indicators (phone, radio, television, meetings, and visits) [51, 52].

### Data sources and sampling

This study applied the purposive sampling technique to administer the questionnaires. Two communities were selected per district, and in each community, one dual moderator focus group discussion and one key informant interviews were held in both Ghana (4) and Zambia (4). The division of role by the two moderators ensured a smooth, focused session to manage the group, and each focus group discussion comprised 10–15 participants [58], with mandatory female and youth inclusiveness because they are essential to the agricultural sector and represent the majority in the population [42, 43].

The questionnaire design assessed the farmers’ perception of agricultural extension services through the effectiveness of PEA training. The questionnaire had three sections: household socioeconomic indicators, agricultural information, and agricultural extension. Three research assistants were recruited and trained for the research and process of administering the questionnaire.

Overall, 10 farmers took a pre-test of the questionnaire to identify any errors, comprehension of the questions, and the time required to complete the task. The correction and adjustments were made to the final questionnaire to improve the quality of data to be collected. The questionnaires were administered in February 2019 in Ghana and March 2019 in Zambia. Only farmers registered at the Ministries of Agriculture as an individual or under a registered agricultural cooperative participated in the survey. Individual farmers’ consent was obtained before administering the questionnaire using a yes/no answer to voluntarily participate in the survey. In Ghana, 120 of 125 administered questionnaires (96%) were completed, and in Zambia, 120 of 130 (92%) were completed. Global Positioning System (Garmin *etrex 30*) coordinates were recorded for mapping the target communities. Other advantages of using the Global Positioning System are giving a precise location and measuring distances to services such as markets, and replicating the study [59]. In both countries, each community was in at least one agricultural operational area, which is covered by one agricultural extension assistant (field staff). It is the smallest administrative unit of agricultural extension services to which an individual agricultural extension assistant can be assigned (it constitutes several villages or settlements).

### Data analysis

An ordered logistic regression analysis was employed to analyze the indicators influencing farmers’ perception toward the effectiveness of the extension services using STATA version 15.0. Similar studies have applied logistic regression and descriptive analysis where the dependent indicator has two outcomes [51, 52, 60, 62, 63]. In this study, ordered logistic regression is ideal because the dependent indicator has multiple outcomes [64] that used the Likert scale to measure farmer’s responses (0 = not effective, 1 = slight effective, 2 = effective, 3 = very effective). A four-point Likert-type scale was used to assess farmers’ perception of the effectiveness of agricultural extension services [62]. The mean of less than 0.5 meant not effective, 0.5–1.4 was slightly effective, 1.5–2.4 was effective, 2.5–3 was very effective. Some of the explanatory indicators were also ordinal, such as the frequency of contact between farmers and extension officers, where a similar mean cutpoint was applied*.* The generalized ordered regression model fits with the odds/parallel-line, logistic regression, and partial proportional odds models [64]. Furthermore, the vital strength of the model is that it supports linear constraints, the computation of probabilities, and survey data estimates [51, 64], as shown by (1):

1 $$P\left({Y}_{i }>j\right)=g\left(X\beta \right)=\frac{\mathrm{exp}(\alpha j+Xi\beta )}{1+\{\mathrm{exp}(\alpha j+Xi\beta )\}}, \quad j= 1,2,\dots , M-1.$$

$M$ is the number of categories of farmers’ perceived effectiveness of agricultural extension services by PEA training in general, given by the ordered dependent indicators (not effective to highly effective). Using the equation above, the probabilities that $Y$ takes on each of the value 1, …, $M$ is equal to

$$P\left({Y}_{i }=1\right)=1-g\left({X}_{i}{\beta }_{1}\right)$$

$$P\left({Y}_{i }=j\right)=g\left({X}_{i}{\beta }_{j-1}\right)-g\left({X}_{i}{\beta }_{j}\right) \quad j=1, 2,\dots ,M-1$$

$$P\left({Y}_{i }=M\right)=1-g\left({X}_{i}{\beta }_{M-1}\right).$$

As $M$ > 2, the model is equivalent to a series of binary logistic regressions, and the categories of the dependent indicators are combined. In this case, for $j$ = 1, category 1 is contrasted with 2, 3, and 4. For $j$ = 2, the contrast is between 1 and 2 against 3 and 4, whereas for $j$ = 3, categories 1, 2, and 3 are against 4. In this model, the $\beta$’s, and not the *α*’s, are equal for all values of $j$ [51, 64].

According to the literature, the regressors for this model include different farmers’ socioeconomic indicators that might affect their perceptions. $\delta$ is the parameter estimate, ${u}_{2j}$ is an error term, and ${u}_{1}$ and ${u}_{2}$ are error terms distributed with mean 0 and variance 1, respectively. At the same time, the farmers’ perception of PEA training methods is the dependent indicator, whereas socioeconomic indicators and access to extensions are explanatory indicators [51, 52, 60–63]. Table 1 shows the explanatory indicators for ordered logistic regressions*.* A three-point Likert-type scale (Table 1) was used to assess farmers’ perception of the impact of agricultural extension services [62]. The mean of less than 0.5 meant no impact, 0.5–1.4 was slight impact and more than 1.5 was impact.

**Table 1:** Description of explanatory indicators in the regression model

Explanatory indicators	Measurement description
Socioeconomic indicators	
Household head age	Number of years
Gender	0 = Female, 1 = Male
Household head education	Number of years attended
Household size	Number of household members
Access to credit	Number of bank/mobile money accounts
Total land size	Area (ha)
Annual agricultural income	Currency ($)
Total livestock	Number livestock owned
Multiple communication indicators	
Owning of cell phones/HH	Number of cell phones
Owning of radios/HH	Number of radios
Owning of televisions/HH	Number of televisions
Frequency of meetings with officer	0 = never, 1 = above 2 months, 2 = monthly, 3 = weekly
Frequency of famer demand for services	0 = never, 1 = above 2 months, 2 = monthly, 3 = weekly
Perceived returns indicators	
Perceived impact on productivity	0 = no impact, 1 = slightly impact, 2 = impact
Perceived impact on adoption rate	0 = no impact, 1 = slightly impact, 2 = impact
Perceived impact on food safety and nutrition	0 = no impact, 1 = slightly impact, 2 = impact

Source: Field survey (2019)

A few common indicators including socio-economic and communications parameters were considered in the analysis like others Birner et al. [8], Swanson et al. [18], Elias et al. [51], Ntshangase et al. [52], Fosu-Mensah et al. [60], Phiri et al. [61], Maoba [62], Asrat and Simane [63] along with key factors identified from the field survey

The results were validated by triangulating the results from the focus group discussion, individual farmer interviews, observations, and literature that was applied to this research [65, 66].

## Results and discussion

### Descriptive indicators

In Ghana, the results show that 74% were male and 26% were female respondents (heads household) (*n* = 120). In Zambia, the results indicate that male respondents accounted for 66% of farmers and female respondents accounted for 34% (*n* = 120). In Ghana, the average age of the respondents was 46.44 years (Table 2), and the average household size was 9.

**Table 2:** Descriptive statistics

Variable	Mean (std. deviation)		*U *test	*p*-value
	Ghana (*n* = 120)	Zambia (*n* = 120)		
Socioeconomic indicators				
Household head age (years)	46.44 (15.96)	44.68 (14.42)	6812.00	0.470
Household size (No)	9.00 (4.00)	5.00 (2.00)	2233.50	0.000**
Household head education (years)	3.15 (5.06)	10.02 (4.00)	2584.00	0.000**
Access to credit (No/Bank account)	0.4 (0.74)	0.71 (1.07)	5985.00	0.008**
Total land size (Ha)	1.51 (10.31)	4.29 (2.97)	2309.50	0.000**
Annual income (Agri and non-agricultural US$)	1520.03 (1932.20)	1801.63 (4809.15)	7005.00	0.717
Total livestock (No)	24.74 (27.67)	84.21 (386.03)	5806.50	0.010*
Multiple communication indicators				
Owning of cell phones (No/HH)	2.51 (1.71)	1.95 (1.52)	5893.50	0.012*
Owning of radios (No/HH)	0.87 (0.90)	0.82 (0.61)	7065.00	0.776
Owning of televisions (No/HH)	0.68 (0.80)	0.62 (0.66)	7058.50	0.771
Frequency of meetings with an officer	0.25 (0.58)	0.21 (0.43)	7062.00	0.708
Frequency of farmer demand for services	1.10 (0.81)	1.13 (0.64)	6773.00	0.369
Perceived returns indicators				
Perceived impact on productivity	1.67 (0.58)	1.57 (0.67)	6724.50	0.269
Perceived impact on the adoption rate	1.38 (0.69)	1.37 (0.73)	7132.50	0.890
Perceived impact on food safety and nutrition	0.68 (0.76)	1.28 (0.79)	4375.00	0.000**

Source: Field Survey (2019)

1GHC = 0.19USD February 10, 2019 (https://www.bog.gov.gh)

1ZKW = 0.083USD March 22, 2019 (https://www.boz.zm/)

Mann–Whitney *U* test *Significant at *p* < 0.05 **Significant *p* < 0.01

In Zambia, the average age of the respondents was 44.68 years, and the average household size was 5. The average number of years in education was 3.15 in Ghana and 10.02 in Zambia. The results show that there is a significant difference (Mann–Whitney *U* test) in the number of years in education (*p* = 0.000), household size (*p* = 0.000), and access to credit (*p* = 0.008). The average size of the land was 1.51 ha in Ghana and 4.29 ha in Zambia. The results show that there is a significant difference in the average land size (*p* = 0.000) and total livestock (*p* = 0.010) between Ghana and Zambia. On multiple communication indicators, the results further reveal a significant difference in the number of cell phones per household (*p* = 0.012). Similarly, on perceived returns indicators, a significant difference is found in the impact on food safety and nutrition (*p* = 0.000) for the two countries.

### Farmers’ perception toward agricultural extension service performance

**Fig. 4:**
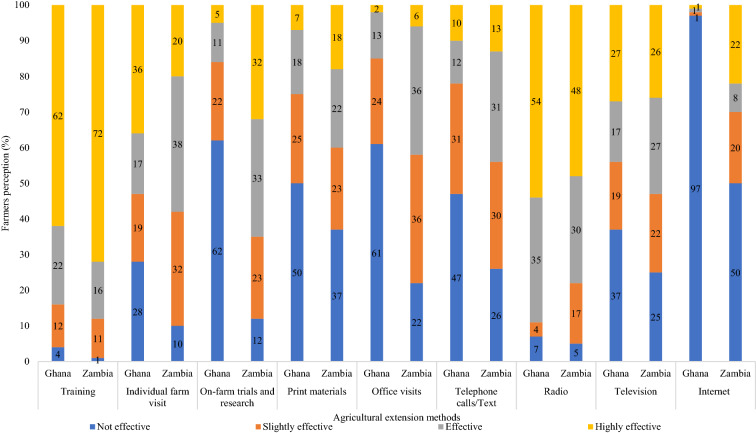
Farmers’ perception of agricultural extension methods through PEA training and selected specific approaches. Source: Field Survey (2019)

In Ghana (*n* = 120), 62% of farmers perceive PEA training as a highly effective agricultural extension approach, compared with 72% in Zambia (*n* = 120) (Fig. 4). Only 4% of farmers in Ghana perceive PEA training as not effective, compared with 1% in Zambia. A detailed analysis of other specific training approaches reveals that 62% of farmers in Ghana perceive on-farm trials and research as not effective, compared with 12% in Zambia. Moreover, 60% of farmers in Ghana and 22% in Zambia perceive office visits as not effective. The use of radio platforms is perceived as highly effective for 54% of farmers in Ghana, compared with 47% in Zambia. We further found that the internet is perceived as not effective by 97% of farmers in Ghana, compared with 50% in Zambia (Fig. 4).

### Influencing indicators toward effective PEA training on extension services

#### Socioeconomic indicators

We found no significant socioeconomic indicators influencing farmers’ perceptions in Ghana (Table 3). However, we found that gender, household size, and access to credit are significant indicators influencing farmers’ perceptions of the effectiveness of training approaches in Zambia (Table 4), comparable with the findings of Elias et al. [51]. The odds of farmers perceiving agricultural training as effective are 2.54 times lower for female farmers compared with their male counterparts (OR 0.222; *p* = 0.011). Although gender is a significant indicator in Zambia, female farmers perceive extension services to be less effective than the male respondents. The gender perception results might suggest that female farmers in Zambia receive poor targeting and access to extension services.

**Table 3:** Indicators influencing farmers’ perception toward the effectiveness of agricultural extension PEA training in Ghana

Explanatory variables	Odds ratio	*z*	*p* > *z*	95% confidence interval	
Socioeconomic indicators					
Household head age (years)	0.984	− 1.190	0.234	0.958	1.010
Gender	1.134	0.250	0.802	0.425	3.023
Household head education (years)	1.025	0.490	0.622	0.927	1.133
Household size (number)	1.123	1.510	0.132	0.965	1.305
Access to credit (Bank accounts)	0.636	− 0.780	0.435	0.204	1.978
Total land size (Ha)	0.897	− 0.460	0.643	0.566	1.421
Annual agricultural income ($)	1.001	1.390	0.165	0.999	1.003
Total livestock (No)	0.986	− 1.570	0.115	0.968	1.003
Multiple communication indicators					
Owning of cell phones (No/HH)	0.457*	− 1.710	0.087	0.186	1.119
Owning of radios (No/HH)	0.798	− 0.650	0.518	0.403	1.580
Owning of televisions (No/HH)	1.351	0.810	0.418	0.651	2.799
Frequency of meetings with officer	0.313**	− 2.510	0.012	0.126	0.774
Frequency of famer demand for services	3.282***	3.350	0.001	1.638	6.573
Perceived returns indicators					
Perceived impact on productivity	2.090*	1.900	0.057	0.978	4.467
Perceived impact on adoption rate	2.911***	2.890	0.004	1.411	6.000
Perceived impact on food safety and nutrition	1.406	1.040	0.298	0.740	2.671
Observations (*n* = 120)	Pseudo *R*^2^ = 0.2239				
	LR Chi^2^ (16) = 54.64				
	Prob > Chi^2^ = 0.0000				

Source: Field Survey (2019)

^*^Significant at *p* < 0.1 **Significant at *p* < 0.05 ***Significant *p* < 0.01

**Table 4:** Indicators influencing farmers’ perception toward the effectiveness of agricultural extension PEA training in Zambia

Explanatory variables	Odds ratio	*z*	*p* > *z*	95% confidence interval	
Socioeconomic indicators					
Household head age (years)	0.998	− 0.12	0.905	0.958	1.038
Gender	0.222**	− 2.54	0.011	0.069	0.710
Household head education (years)	1.070	1.00	0.315	0.938	1.219
Household size (number)	1.383**	2.10	0.036	1.021	1.872
Access to credit (Bank accounts)	5.215**	2.09	0.037	1.107	24.553
Total land size (Ha)	1.012	0.12	0.901	0.843	1.213
Annual agricultural income ($)	1.000	0.08	0.934	0.999	1.000
Total livestock (No)	1.000	0.00	0.997	0.997	1.002
Multiple communication indicators					
Owning of cell phones (No/HH)	0.572	− 1.04	0.300	0.198	1.646
Owning of radios (No/HH)	0.587	− 1.02	0.306	0.211	1.627
Owning of televisions (No/HH)	0.234**	− 2.25	0.024	0.066	0.827
Frequency of meetings with officer	0.290*	− 1.71	0.087	0.070	1.195
Frequency of famer demand for services	2.194*	1.75	0.081	0.908	5.298
Perceived returns indicators					
Perceived impact on productivity	2.417**	2.13	0.033	1.073	5.442
Perceived impact on adoption rate	2.323**	2.20	0.028	1.096	4.920
Perceived impact on food safety and nutrition	2.391**	2.14	0.033	1.075	5.316
Observations (*n* = 120)	Pseudo *R*^2^ = 0.256				
	LR Chi^2^ (16) = 49.39				
	Prob > Chi^2^ = 0.000				

Source: Field Survey (2019)

^*^Significant at *p* < 0.1 **Significant at *p* < 0.05 ***Significant *p* < 0.01

Likewise, the odds of farmers perceiving agricultural training as effective are 2.10 times greater for each unit increase in household size (OR 1.383; *p* = 0.036). The average household size in Ghana is 9, whereas, in Zambia, it is 5. Every increased unit in Zambia is an essential indicator influencing farmers’ perception, as compared with that in Ghana. Larger household sizes are vital for farmers to adopt labor-intensive agricultural practices and diversify into non-farm activities for income generation [67]. A higher level of education is critical because it enables farmers to read, write, and comprehend more information during training, compared with those with less education.

Controlling for other variables, the odds of perceiving agricultural training as effective by farmers with access to credit are 2.09 times greater than those by farmers without access to credit (OR 5.215; *p* = 0.037). Furthermore, we found a contradiction in Hamilton and Hudson [68] that access to credit advice has a limited positive impact on crop yield and income of farmers in Ethiopia. Our results in Zambia show a positive influence on farmers’ perception of the effectiveness of extension services. However, although insignificant, their findings are consistent with our results from Ghana. Access to credit is key to adopting technologies and practices that require investment [69] and could affect perception because farmers can use agricultural information. Using data from wheat farmers in Harayana, India, Coventry et al. [70] have demonstrated that socioeconomic indicators are critical to identify farmers’ efficiency in using agricultural information and technology.

#### Multiple communication indicators

The multiple communication indicators considered in this study are ICT and physical interaction between agricultural extension officers and farmers. The difference in the indicators influencing farmers’ perception of the effectiveness of training approaches is that the number of cell phones in a household is significant in Ghana but not in Zambia. The ordered regression results show that the number of cell phones, meetings, and frequency of farmer demand for services are statistically significant in Ghana (Table 3). The odds of farmers perceiving agricultural training as effective are 0.457 times greater for every unit of cell phone less per household (OR 0.457; *p* = 0.087). Contrastingly, the number of televisions was significant in Zambia. The odds of a farmer perceiving agricultural training as effective are 2.25 times greater for every unit of television less per household (OR 0.234; *p* = 0.024).

Alternatively, the findings of Elias et al. [51] show no significance for multiple communication indicators. However, Moussa et al. [71] demonstrated that in Niger and Burkina Faso, the reinforcement of extension programs with ICT, such as radio messages, yields better results regarding the adoption of the triple bagging technology for cowpea. In West Macedonia, Greece, Anastasios et al. [72] also found that using technologies will supplement the traditional methods rather than replace them.

The multiple communication indicators significantly influencing farmer’s perception in both Ghana and Zambia are meetings with extension officers and farmers’ demand for extension services. In Ghana, the odds of perceiving agricultural training as effective by farmers who irregularly attend meetings are 0.313 times lower than those by farmers who regularly attend meetings (OR 0.313; *p* = 0.012). However, the odds of perceiving agricultural training as effective by farmers who regularly demand agricultural services are 3.350 times greater than those by farmers who rarely demand the services (OR 3.282; *p* = 0.001). In Zambia, the odds of perceiving agricultural training as effective by farmers who irregularly attend meetings are 1.71 times greater than those by farmers who regularly attend (OR 0.290; *p* = 0.087). Furthermore, the odds of perceiving agricultural training as effective by farmers who regularly demand for agricultural services is 1.75 times greater than those by farmers who irregularly demand for the services (OR 2.194; *p* = 0.081).

These results, meetings, and demand for service indicators are consistent with farmers’ perception of adopting no-till tillage in South Africa [52], satisfaction with extension services in Ethiopia [51], and adaptation to climate change in Ghana [59]. Equally, Joshi and Narayan [73] found that the frequency of extension contact is a significant indicator in the performance measurement of agricultural extension in sustainable livelihoods in India.

These results suggest that there is a need to improve the interaction of agricultural extension officers and farmers to enhance the performance of agricultural extension services. The participatory interactions tap into farmers’ knowledge, men, women, and youths and are vital for effective agricultural extension services [74, 75]. Participatory extension approaches are vital for technology adoption, farmer-to-farmer extension, and sustainable local solutions [74–77]. Successful examples of participatory agricultural extensions include the increasing diversity of plant biomass in semiarid Burkina Faso; farmers, extension, and research partnerships in Tanzania; and technology development for soil fertility improvement in Cameroon [74]. Chris and Waters-Bayer [74] further highlight some case studies such as mainstreaming participatory approaches in soil and water conservation in Zimbabwe and learning sustainability through participatory approaches for rural development in Ethiopia and Tanzania. Studies have also shown that on-farm interaction increases the effectiveness of agricultural extension in Swiss farmers on the implementation of ecological compensation on their farms [78].

#### Perceived returns indicators

In Ghana and Zambia, perceived economic returns indicators such as productivity and adoption rate significantly influence farmers’ perceptions, consistent with the findings of Elias et al. [51] on farmers’ satisfaction with extension services in Ethiopia. In Ghana, the odds of farmers perceiving agricultural training as effective in affecting productivity are 1.900 times greater than those of farmers who perceive no effect (OR 2.090; *p* = 0.057). Similarly, the odds of farmers perceiving agricultural training as effective in affecting the adoption rate of technologies are 2.890 times greater than those of farmers who perceive no effect (OR 2.911; *p* = 0.004). In Zambia, the odds of farmers perceiving agricultural training as effective in affecting productivity are 2.13 times greater than those of farmers who perceive no effect (OR 2.417; *p* = 0.033). Similarly, the odds of farmers perceiving agricultural training to be effective in affecting the adoption rate of technologies are 2.20 times greater than those of farmers who perceive no effect (OR 2.323; *p* = 0.078). Equally, the odds of farmers perceiving agricultural training as effective in affecting food safety and nutrition are 2.14 times greater than those of farmers who perceive no effect (OR 2.391; *p* = 0.033). The impact of training on food safety and nutrition is essential for alleviating undernutrition and overnutrition (obesity), which are prevalent in both developed and developing countries [79, 80].

The results also suggest that agricultural extension services training should include food safety and nutrition to achieve a maximum impact within the food system [79–82]. Tuholske et al. [83] found that 70% of Ghanaian households’ could experience challenges in having adequate food, resulting in poor nutritional status of such families [84]. Schmidhuber and Shetty [79] argue that food safety and nutrition is a significant double burden that needs urgent attention in developing countries such as Ghana and Zambia. Evidence of the importance of food nutrition has been highlighted by the current Coronavirus (COVID-19) pandemic [85, 86]. Experts suggest that those with poor nutrition (diets) are significantly affected and will have long-term effects if they recover [87] and redesign of the food system [85–89]. Hence, private–public partnerships, institutional collaboration, and policy integrations are some of the potential solutions on food and nutrition that required strengthening and harmonization [79–82].

### Farmers’ opinion on effective PEA training

The farmers’ perception of agricultural extension training as highly effective is lower in Ghana (62%) than that in Zambia (72%). Agbarevo and Benjamin [90] found an even lower perception of 39.65% in Cross River State, Nigeria, whereas Ali et al. [91] found no significant difference in the effectiveness of agricultural extension between farmers who receive extension services and the control group in Jordan. In Punjab Province, Pakistan, Davidson and Ahmad [24] found that both the private and government extensions are not effective in meeting the expectation of cotton farmers. Kumaran [92] showed that aqua farmers’ perceptions in two Indian states—Andhra Pradesh and Tamil Nadu—indicated that public extension services required improvement, whereas private agricultural extension was effective. The collaborations could be of significant value in providing better agricultural extension services to most small-scale farmers.

On the socioeconomic front of farmers in Ghana, despite gender not being a significant indicator, with similar finding by Danso-Abbeam et al. [93], female farmers are more likely to perceive extension services as effective, and women account for 43% of the agricultural labor force in developing countries [94]. According to FAO [94], female participation in agriculture could increase agricultural productivity by 20–30%, increase national agricultural outputs by 2.5–40%, and save 100–150 million people from hunger in all. For instance, in Ghana, despite women not owning the land, they contribute significantly to agriculture. “As women, we are actively involved in farming, trading in agricultural produce. We do the processing of groundnuts into butter, process shea butter, and brew local drink using sorghum (*Pito*)” (Chanssa community, Female, Interview, 2019).

**Fig. 5:**
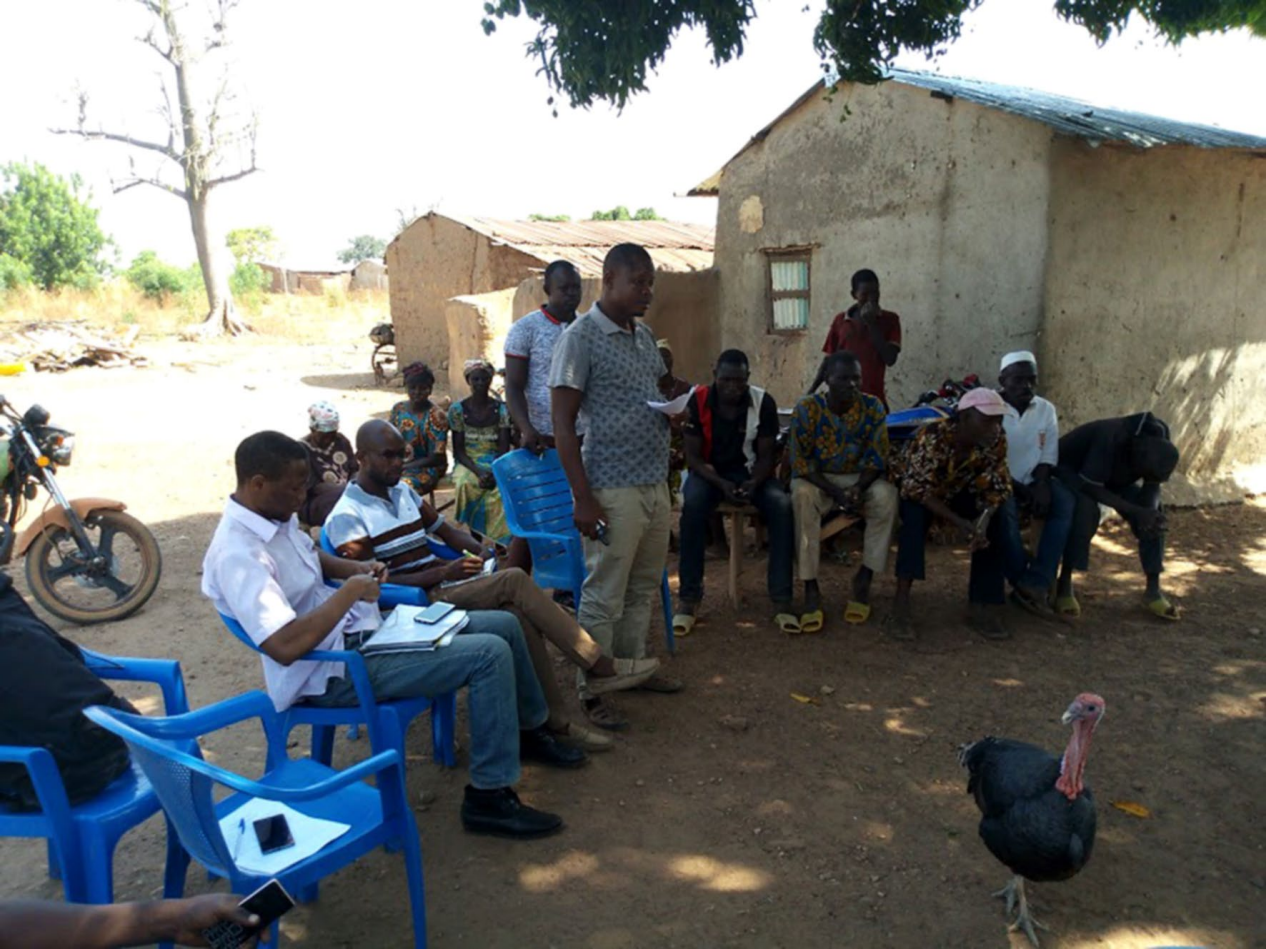
Focus group discussion in Sagu community, Wa East District, Ghana. Source: Field Survey (2019)

Our results show that multiple communication indicators, such as meetings, have negative significance because most farmers rarely attend them. For example, during the discussions, in Ghana (Fig. 5), it was revealed that farmers are less likely to have meetings with the extension officer because they are not present in the communities.

**Fig. 6:**
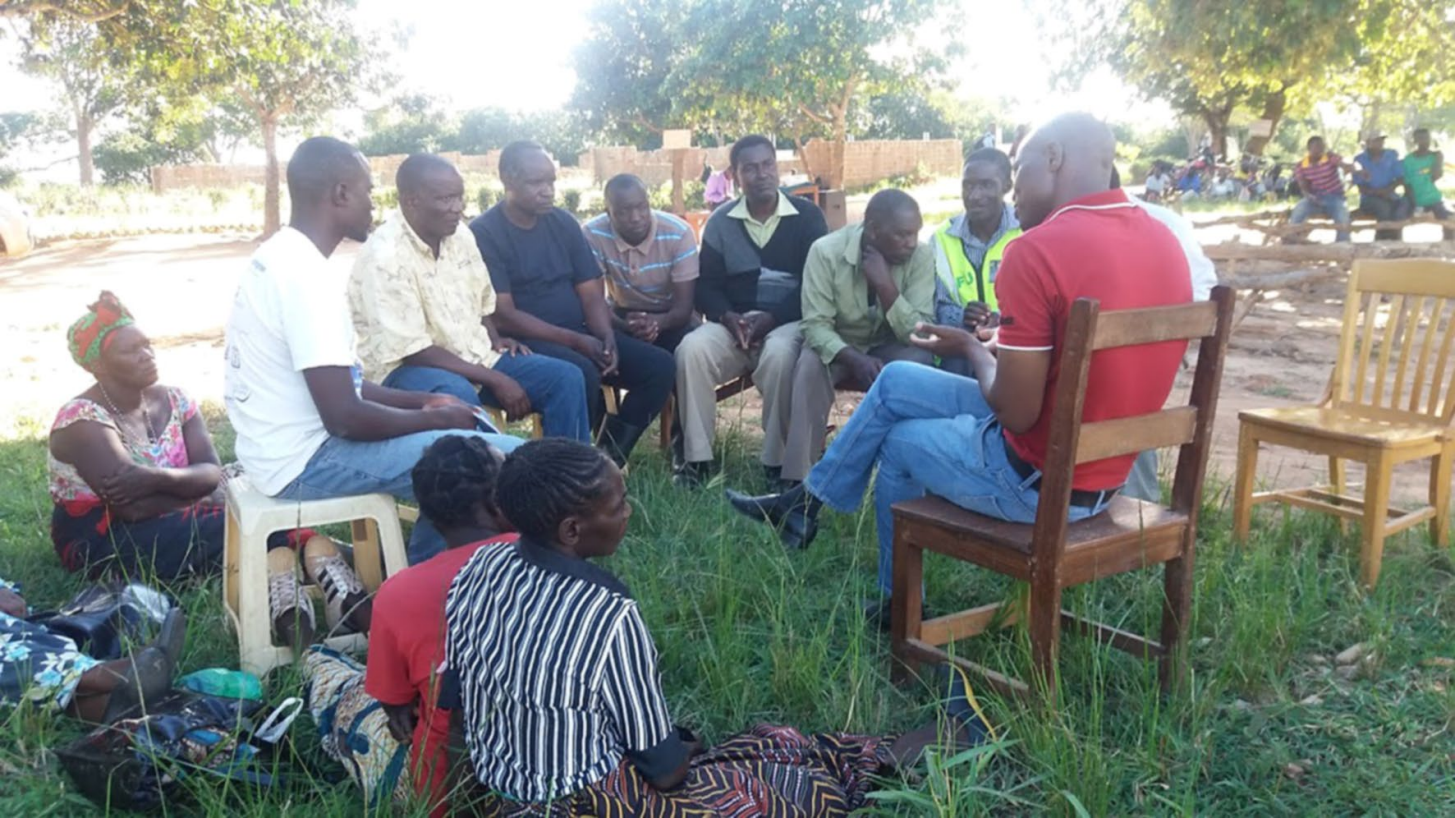
Focus group discussion in Muzoka community, Pemba District, Zambia. Source: Field Survey (2019)

“Extension officers are rarely seen here because they stay in Wa town, and they have not moved the offices to Wa East District here … they are still operating from Wa district” (Sangu community, Male, Interview, 2019). In Zambia, during the discussions (Fig. 6), farmers reported that officers only call a meeting if they need some information from them. “Agricultural extension officers claim they don’t have fuel and transport for the regular meeting. We have to follow them at their offices or homes if we need some agricultural information from them” (Siamuleya community, Male, Interview, 2019).

However, there are positive sentiments from farmers on the perceived returns of agricultural extension training. Our discussion with farmers confirmed that they are adopting sustainable practices to cope with regular droughts. “Some sustainable practices the farmers are practicing are early planting, early maturing varieties, ripping, manure application, liming and crop rotation/diversification” (Muzoka community, Male, Interview, 2019).

## Conclusions with policy implications and future works

In this study, we found that 62% and 72% of farmers perceive PEA training as a significantly effective agricultural extension approach in Ghana and Zambia, respectively. These results could further be improved by adopting specific training approaches based on the different farmers’ characteristic indicators, such as owning cell phones, as shown by the evidence from Ghana. Gender, household size, access to credit, owning a television, and food safety and nutrition are significant indicators in Zambia. Both Ghana and Zambia need to increase interaction (regular meeting and demand for services), including using local radio/television stations and phones (ICT-based approaches) between agricultural extension service providers and farmers to enhance farmer participation and adoption of sustainable agricultural practices. Specifically, using cell phones in Ghana and television in Zambia, as shown by the results, are significant and guided by the research framework. In addition, we demonstrated that some indicators are more significant in one country than the other, whereas others are similar. Furthermore, food safety and nutrition as an indicator is vital for assessing the effectiveness of agricultural extension service performance.

We identified the following four policy implications relevant to enhancing agricultural extension performance.

1. Enhancing public–private partnerships in the delivery of agricultural extension services. The collaboration with multimedia platforms (print, radio, television, and cell phone), agricultural research, and technology providers could be of significant value in providing better pluralistic agricultural extension services to most small-scale farmers.
2. The significant socioeconomic indicators should be considered as entry points to target specific farmers in enhancing their productivity and improving the effectiveness of agricultural extension performance. For example, extension performance could be improved by increasing the participation of women, focusing on farmers with less education, and enhancing access to credit for farmers to improve the adoption rates of technologies. Similarly, a positive influence is possible using better-performing farmers as role models and community-based extension agents for farmer-to-farmer extension services.
3. Appropriate agricultural extension communication methods should be used, depending on their significance, to influence farmers’ decisions and improve the effectiveness of the extension services. Targeted and multiple communication approaches, such as the integration of ICT and participatory agricultural extensions, are more effective than a single blanket extension method for all farmers. Improving the least perceived approaches, such as using the internet and printed material, should also be emphasized.
4. The focus of agricultural extensions should be on agricultural production and marketing processes, including a vital component of food safety and nutrition, to enhance human health and well-being toward changing production and consumption patterns.

Methodologically, despite the evidence provided in this study by measuring intangibles using perception, when it is challenging to measure some indicators empirically, there might be bias, which is a limitation of this study. In this study, 74% and 66% of the respondents were males in Ghana and Zambia, respectively. Future studies should consider using longitudinal research and balancing the gender of the respondents to enhance the results. Further research on the empirical monitoring mechanisms of proposed policies and the efficiency of the pluralistic agricultural extension performance is equally important.

## Data Availability

The data set for this study is available from the corresponding author on if requested.

## Abbreviations

COVID-19: Coronavirus
CSO: Central Statistical Office
FAO: Food and Agricultural Organization
GSS: Ghana Statistical Services
ICT: Information Communication and Technology
IFPRI: International Food Policy Research Institute
MoA: Ministry of Agriculture
MoFA: Ministry of Food and Agriculture
PEA: Participatory Extension Approach
SDGs: Sustainable Development Goals
SSA: Sub-Saharan Africa
UNDESA: United Nations Department of Economic and Social Affairs
